# Design, Stereolithographic 3D Printing, and Characterization of TPMS Scaffolds

**DOI:** 10.3390/ma17030654

**Published:** 2024-01-29

**Authors:** Roberta Gabrieli, Raphael Wenger, Marco Mazza, Enrica Verné, Francesco Baino

**Affiliations:** 1 Institute of Materials Physics and Engineering, Department of Applied Science and Technology, Politecnico di Torino, 10129 Torino, Italy; roberta.gabrieli@polito.it (R.G.); enrica.verne@polito.it (E.V.); 2 School of Engineering and Architecture Fribourg, University of Applied Sciences and Arts Western Switzerland, 1700 Fribourg, Switzerlandmarco.mazza@hefr.ch (M.M.)

**Keywords:** biomaterials, scaffold, additive manufacturing, triply periodic minimal surface, tissue engineering

## Abstract

Anatomical and functional tissue loss is one of the most debilitating problems and involves a great cost to the international health-care sector. In the field of bone tissue, the use of scaffolds to promote tissue regeneration is a topic of great interest. In this study, a combination of additive manufacturing and computational methods led to creating porous scaffolds with complex microstructure and mechanical behavior comparable to those of cancellous bone. Specifically, some representative models of triply periodic minimal surfaces (TPMSs) were 3D-printed through a stereolithographic technique using a dental resin. Schwarz primitive and gyroid surfaces were created computationally: they are characterized by a complex geometry and a high pore interconnectivity, which play a key role in the mechanism of cell proliferation. Several design parameters can be varied in these structures that can affect the performance of the scaffold: for example, the larger the wall thickness, the lower the elastic modulus and compressive strength. Morphological and mechanical analyses were performed to experimentally assess the properties of the scaffolds. The relationship between relative density and elastic modulus has been analyzed by applying different models, and a power-law equation was found suitable to describe the trend in both structures.

## 1. Introduction

In recent years, the sudden growth in the field of material manufacturing technologies has opened up new possibilities in many industrial, medical and high-tech sectors, leading to great improvements in the relevant areas. The development of additive manufacturing approaches—also commonly called 3D printing—allows the fabrication of 3D objects through layer-by-layer production, starting from a virtual model that may be created by means of specific software [1], and 3D printing is highly appealing in the medical field. It was first introduced in 1990 in the dental sector, and today is also used in the development of medical equipment and the creation of customized devices to improve the accuracy of surgical procedures.

Nowadays, the demand for implantable devices and artificial organs is increasing considerably, mainly due to the increase in the life expectancy and aging population worldwide. Hence, a new discipline called “tissue engineering” has emerged [2] that, after its introduction at the first NSF congress in 1988, identifies “the set of principles and methods of the medical and engineering sciences to establish the fundamental relationships between structure and function of healthy or pathological mammalian tissues and the development of biological substitutes to restore, maintain or improve tissue function” [3]. In tissue engineering, 3D printing can play a key role, as it shows promise for creating new implantable tissue and devices reliably and potentially at an affordable price.

Bone tissue is the second-most frequently transplanted tissue after blood [4], and thus the development of tissue-engineered constructs for osseous replacement is of high interest in our society. When bone tissue needs to be repaired due to severe pathology or trauma, an innovative therapeutic approach relies on implanting a porous scaffold that serves as a 3D template for the growth of new healthy tissue.

The scaffold is a porous structure, based on natural or manmade materials, that is typically created to mimic the architecture of the tissue that needs to be replaced and enables cell adhesion, proliferation and differentiation, thus stimulating tissue growth [5,6]. It can be designed as a permanent implant or a temporary 3D guide for the growth of new tissue, which will safely dissolve once it has performed its function. Hence, a very important step in the production of a scaffold lies in the choice of its architecture as well as the biomaterial for making it. Scaffolds must also positively influence the mechanical and biological properties of the tissue and cells [7]. Bone tissue-engineering scaffolds should be biocompatible, bioactive, bioabsorbable without releasing any toxic by-products, osteoconductive and osteoinductive, exhibit adequate mechanical properties to withstand loads, and have an appropriate architecture in terms of pore volume and pore size [8].

The main properties and the architecture of bone should be carefully considered at the design stage of scaffolds for bone tissue engineering [9]. In particular, the scaffold should mimic the key properties of the human trabecular bone, characterized by high pore interconnectivity, porosity between 40 and 90 vol.%, compressive strength of 2–12 MPa and Young’s modulus of 0.05–0.5 GPa [10,11].

The success of the scaffold depends both on the biomaterial chosen for its production and on the method used to produce it. The materials commonly used for making scaffolds for bone tissue engineering include polymers, crystalline (e.g., hydroxyapatite) and amorphous ceramics (e.g., bioactive glasses), and composites. Porous metallic implants have also been proposed, mainly due to their high mechanical properties, but they typically show poor osteointegration and carry the risk of in vivo corrosion and release of toxic ions [12,13].

Each class of material is characterized by advantages and limitations for bone-contact application. As regards organic substances, both natural and synthetic polymers are used in bone tissue engineering applications [14]. Natural polymers are biocompatible, osteoconductive and bioactive, and provide great cell attachment and growth. The main advantages of these materials include controlled biodegradability and low immunogenic potential, while they suffer from poor mechanical properties and poor stability. Synthetic polymers have inferior bioactivity compared to natural ones, but on the other hand, they have stronger mechanical properties and can be easily fabricated with a tailored structure and in different shapes [7,8,15].

Bioceramics are widely used in bone tissue engineering due to their high biocompatibility, bioactivity (i.e., bone-bonding ability) and physicomechanical similarity with hard tissue [16]. Bioceramics also exhibit high corrosion resistance and are strong in compression, but mechanically weak under tensile/flexural loads.

Composite materials are made up of a combination of single-phase components to achieve the best characteristics for bone scaffolds. Composite biomaterials typically comprise a polymeric matrix, providing softness, pliability and controlled degradability, in which a stronger, bioactive inorganic phase is dispersed [17].

Different methods have been developed to fabricate 3D bone scaffolds, which are divided into conventional techniques and additive manufacturing strategies. Conventional techniques mainly rely on the use of (i) solvents and solutes in liquid or non-liquid form for the production of polymeric or composite scaffolds, or (ii) sacrificial templates for obtaining porous bioceramics. Additive manufacturing technologies can overcome the limitations of conventional techniques (e.g., poor reproducibility and scalability, poor control on internal features) and create the desired structure of the scaffold through layer-by-layer production [15].

These 3D printing techniques have become increasingly popular for bone scaffolding, and encompass inkjet printing, laser/light-assisted strategies and extrusion-based printing; in general, all of them allow the creation of scaffolds with complex internal geometries and architectures, such as bone-like structures [12]. The unit cell of 3D-printed scaffolds, initially of non-parametric type, has been replaced over the years with a parametric type. The unit cell for a non-parametric scaffold consists of a structure with simple geometry, such as a circular or cubic geometry, to obtain a simple grid. Through the use of appropriate algorithms, it is possible to obtain parametric unit cells that have complex geometries.

With the aim of imitating natural pore structures and fulfilling demands in many engineering applications, triply periodic minimal surfaces (TPMSs) have attracted increasing interest over the last few years. For example, TPMS structures are used for thermal and electrical insulation [18] and in structural engineering applications [19,20], and they have also been recently claimed as highly promising in bone tissue engineering [21].

The TPMS is defined mathematically as a surface that self-repeats in 3D with a large surface area. It is characterized by a minimal surface because it has a zero-mean curvature over its entire surface, which makes it similar to bone trabecular structures [22]. Moreover, TPMS structures have a smooth surface, which mechanically leads to a reduced stress concentration [23,24] and exhibit an open cell network that provides continuous porosity for vascularization and highly interconnected pores that can supply adequate space for cell attachment [25,26]. Olivares et al. [27] demonstrated the higher capability of gyroid surfaces compared with conventional hexagonal structures to promote the cell differentiation process. Gyroid architecture and pore distribution provided better accessibility of the fluid than hexagonal architecture. A similar study conducted by Al-Ketan et al. [28] analyzed the mechanical performance of TPMS-based structures and strut-based structures, and it was shown that the TPMS-based structures exhibited superior mechanical properties.

Generating a structure with similar features to a natural porous architecture is the challenge of TPMS design. In addition to being expressed through simple mathematical functions, TPMS structures allow management of their performance simply by acting on some of their geometrical parameters, such as overall architecture, curvature range, periodicity, total porosity, and specific surface area, which can all be easily controlled.

Scaffolds for bone tissue engineering should fulfill important requirements in terms of mechanical performance, which in turn, in TPMS structures, is linked to and influenced by the relative density of the porous structure [29].

Commonly used TPMS structures are Schwarz primitive, diamond, and gyroid. Several researchers have investigated the mechanical and geometrical properties of TPMS structures for potential use in bone tissue engineering. Wang et al. [30] designed several primitive-based scaffolds that were then fabricated by selective laser melting, and showed that the mechanical properties and permeability of the scaffolds were actually suitable for use in bone tissue replacement. Zhu et al. [31] designed scaffolds with optimized structures also possessing mechanical properties and permeability suitable for bone tissue applications. Peloquin et al. [32] investigated the volumetric accuracy, printability and mechanical behavior of different 3D-printed photopolymer lattice structures. Their study evaluated an extensive range of lattice porosities produced by stereolithography, and the results demonstrated the mechanical superiority of the gyroid lattice compared to the diamond lattice for all measured porosities and materials.

The present study is focused on the design and fabrication of two TPMS structures, gyroid and Schwarz primitive, using an optically transparent dental resin. TPMSs were first described using an implicit method through a trigonometric function. Implementation was performed in Python, which makes the visualization of TPMSs and the change in relevant parameters easy. Then, polymeric scaffolds were experimentally fabricated by stereolithography, which allows printing complex TPMS structures with high resolution and reproducibility. The scaffolds were finally characterized in terms of mechanical performance and a qualitative analysis performed according to ISO standards and relevant modeling. To the best of the authors’ knowledge, this is the first work dealing with the production and comparison of two different TPMS-type scaffolds using the commercial Dental LT clear resin, which is commonly used for medical purposes. Looking at the previous literature, only one study was found, where Karillova et al. [33] produced gyroid scaffolds using Dental LT clear resin by stereolithography and compared them with analogous structures obtained by using different materials and additive manufacturing techniques. However, that study was not addressed to assess the mechanical properties of the specific TMPS geometry, but to develop a strategy to create biomedically relevant composite scaffolds using a bioceramic-based bone adhesive. Here, we also investigated the relationship between relative density (porosity) and mechanical properties, which is important in the frame of optimal scaffold selection.

## 2. Materials and Methods

### 2.1. Design of Scaffolds Based on TPMSs

Scaffold geometries were designed using Schwarz primitive (Schwartz P) and gyroid surfaces, which can be expressed in an implicit way as trigonometric functions:

Schwarz P:

Fp = cos(*αx*)+ cos(*αy*) + cos(*αz*) = C, (1)

Gyroid:

Fg = sin(*αx*) cos(*αy*)+ sin(*αy*) cos(*αz*) + sin(*αz*)cos(*αx*) = C, (2)

All the parameters were set in Python (Python Software Foundation, Wilmington, DL, USA, version 3.8) to create different elementary cells. The trigonometric function describes a constant-level isosurface set by C; specifically, the functions Fp or Fg determine the surface topology and C is the level set parameter.

**Figure 1:**
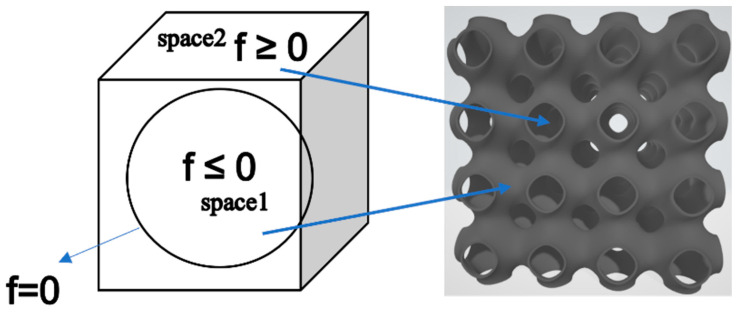
Subdomains of the 3D volume.

C controls the expansion or contraction of the surface in three spatial directions and influences the density of the lattices [34]. It can assume constant value or be dependent on the spatial coordinates *x*, *y*, and *z*. If C is a linear or quadratic function of *x*, *y* or *z*, it describes a surface with different porosity in the three directions. A porosity gradient is thus introduced where the pores vary in size according to the specific coordinate value (graded scaffold). When the equation is equal to zero, i.e., C = 0, the isosurface divides the 3D space into two subdomains with the same volume (Figure 1). The part where the trigonometric function is negative or equal to zero is defined as the portion occupied by the surface, while the region where the function is greater than zero is defined as empty space [35].

**Figure 2:**
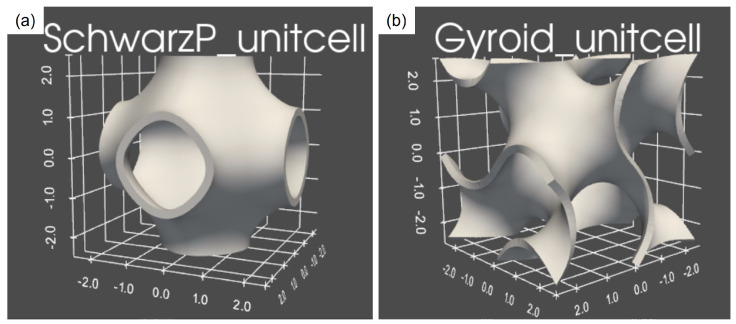
Representation of solid unit cell: (**a**) Schwarz P type; (**b**) gyroid type.

The parameter a is equal to 2π/L, where L is the length of the unit cell and is used to define the periodicity of the cell, which determines the size of the lattice. In order to transform the surface into a 3D structure, a thickness is introduced using the Blender functions in Python to control and vary its size. The representation of the gyroid and Schwarz P solid unit cell created in Python is shown in Figure 2.

### 2.2. Scaffold Fabrication

The 3D models were triangulated using contouring algorithm and then exported as STL files as required by the printer software. Stereolithography was selected as the additive manufacturing method for scaffold fabrication due to its great versatility and the previous experience of the authors with this technology [36,37].

All scaffolds were fabricated by the stereolithographic 3D printer Form2 (Formlabs Inc., Somerville, MA, USA) using a biocompatible commercial resin (Dental LT Clear Resin, Formlabs Inc., Somerville, MA, USA) as a printable material. It is a transparent resin, so it was possible to analyze the internal structure of scaffolds by optical microscopy. It is also characterized by high fracture resistance and good long-term wear resistance [38].

The Preform software (Formlabs Inc., Somerville, MA, USA) allows for setting the model orientation, position and dimensional scale before the printing process. Finding an optimal orientation of the sample and using a support structure were recommended to reduce the risk of print failure. For example, tilting of the sample was recommended to reduce contact between the tank and the workpiece, which could result in a greater force to the product being printed whenever the platform was raised [39].

Form2 printer works with liquid thermosetting resins, which react and polymerize (solidify) when exposed to light with a wavelength of 405 nm. The process was carried out at room temperature with automatic printing temperatures of around 30–35 °C.

All fabricated samples were washed with Form Wash using isopropyl alcohol. Uncured resin was removed from the sample surface using a propeller that moves the alcohol to reach the inside of the workpiece. Post-processing included photopolymerization in the Form Cure to maximize material performance. The Form Cure chamber was heated to 60 °C, and 13 multidirectional LEDs inside triggered the post-curing reaction. In order to do that, the printed piece was placed on a turntable that provided uniform light exposure for 60 min.

### 2.3. Materials

The material selected for scaffold fabrication was Dental LT clear resin (Formlabs Inc., Somerville, MA, USA), which is clinically used for occlusal splints, aligners, palatal plates, long-term printed orthodontic devices and also in orthopedic appliance components [40].

This resin is classified as IIa biocompatible material, and is recommended for long-term use, is safe and affordable, and is also suitable for mucosal and skin surfaces [40,41,42]. This resin fulfils the essential requirements of the EU Council Directive 93/42/EEC as well as some EN-ISO standards. The full material properties and safety data are available in [43]. The resin density reported in the safety datasheet is 1.1–1.2 g/cm^3^, and the ultimate flexural strength reported in the technical datasheet is >50 MPa. This value is also consistent with the compressive strength (60 MPa) that was experimentally calculated in the present study using a non-porous cube of resin.

Dental LT clear resin has been tested in several studies in order to assess its performance. A study conducted by Jindal et al. [44] compared the mechanical and geometrical properties of 3D-printed Dental LT clear resin and conventionally manufactured thermoformed Dental clear aligners, reporting that the former had sufficient mechanical strength and overall characteristics comparable to the thermoplastic materials. Concerning the mechanical properties, Milovanovic et al. [45] evaluated the tensile, compressive, flexural strength and strain at failure of the Dental LT clear resin over time. The results indicated that the resistance to tensile, compressive and bending loads increased with time, while the elongation at failure was reduced. This research also confirmed that 3D-printed aligners could satisfy the demand of orthodontic applications. A study by Paradowska-Stolarz et al. [46] demonstrated that Dental LT clear resin remained stable from a mechanical viewpoint when tested under compression.

This resin also shows good aesthetic properties, transparency/translucency, resistance to discoloration over time, and biocompatibility in the long term [46,47,48].

### 2.4. Case Studies

In this work, two different TPMS basic structures, Schwarz P and gyroid, were designed in Python and subsequently analyzed. Both surfaces had a C value equal to 0 and a constant wall thickness. They were chosen in order to have full interconnectivity of pores (no closed pores). All the structures and derived scaffolds had cubic shape. The theoretical volume of scaffolds was 12 × 12 × 12 mm^3^; accordingly, some scaffolds were obtained by replicating basic structures with a unit cell length of 3 mm four times in 3D, while others were obtained by replicating basic structures with a unit cell length of 4 mm three times in 3D. A wall thickness from 0.1 mm to 0.5 mm, in 0.1 mm steps, was chosen for each unit cell length. The parameters set in Python are shown in Table 1.

For each scaffold designed in Python, the porosity and the relative density were calculated through the following formulae:

percentage total porosity = (pore volume)/(total volume) × 100(3)

percentage density = (solid volume)/(total volume) × 100(4)

Theoretical porosity calculations were done by Python. The total volume was the volume of the cube that included both the void spaces (pores) and the solid part; the pore volume was calculated for the difference between the total volume and the volume occupied by the surface (solid skeleton). The analysis of density and porosity is important because the mechanical resistance, the durability of the scaffold, and the cell proliferation depend on it [49]. TPMS scaffolds designed in this work had a theoretical porosity ranging from 50 to 98 vol.%.

### 2.5. Scaffold Characterizations

The parameters set in Python were expected to affect the mechanical and morphological properties of each scaffold.

The bulk density was experimentally calculated through mass and volume measurements for every individual scaffold. Mass was assessed through using a balance with an accuracy of 10^−4^ g and a maximum capacity of 510 g; the volume was calculated by making the product between the height, width and length of each individual sample (cuboid). In particular, the measures of length, width, and height were obtained by averaging, for each size, three measurements that had been taken through a caliper.

The total porosity (vol.%) of each scaffold was experimentally determined from the relative density (i.e., the ratio between bulk density ρ_s_ and the density of non-porous material ρ_0_) as 100 × (1 − ρ_s_/ρ_0_). The value of ρ₀ was assessed by mass–volume measurements on a non-porous cuboid made of the same resin used to print the scaffold.

Scaffold morphology was inspected by digital optical microscopy (KEYENCE VHX-6000, Keyence Corporation, Osaka, Japan) and scanning electron microscopy (SEM; JCM-6000Plus benchtop SEM, JEOL, Tokyo, Japan).

The mechanical properties of scaffolds were determined by compressive tests using a Shimadzu Autograph AGS-X machine (Shimadzu, Kyoto, Japan) with a 10 kN load cell and loading rate of 1 mm/min, following the recommendations provided by ISO 604:2002 standard [50]. The force was applied orthogonally to the specimen section. Fixed-type compression plates with a diameter of 50 mm and a thickness of 25 mm were used for the compressive test. Cubic scaffolds were positioned in the center of the plates, and 5 samples were tested for every TPMS scaffold type. The recorded force and displacement were used to obtain the stress–strain curve from each compressive test. Young’s modulus and compressive strength (i.e., yield stress in this work) were calculated directly from the stress–strain compressive plots. The elastic modulus was determined from the linear region of the curve. Results are expressed as average ± standard deviation calculated for five specimens for every TPMS scaffold type.

## 3. Results and Discussion

### 3.1. Geometry and Morphology of Scaffolds

**Figure 3:**
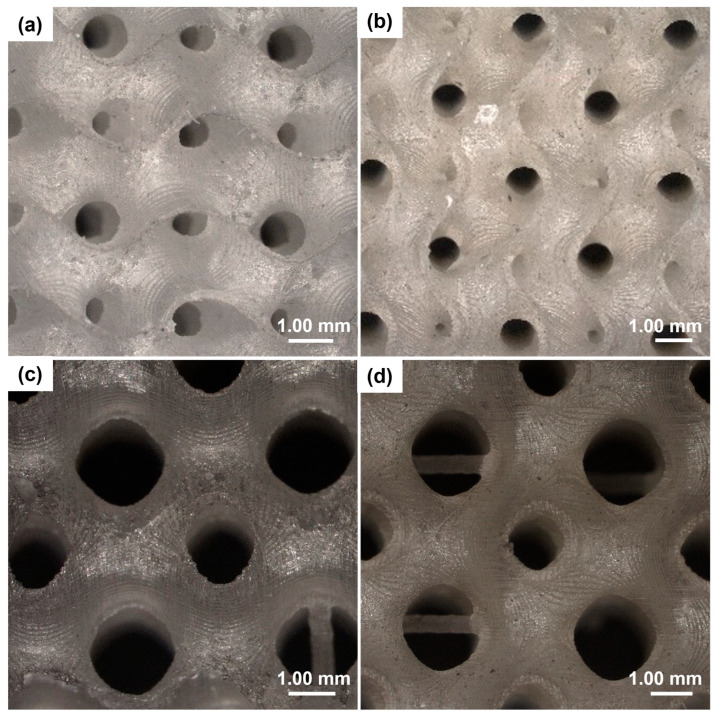
Optical microscopy images of TPMS scaffolds with a unit cell length of 4 mm. (**a**) Gyroid with thickness of 0.1 mm. (**b**) Gyroid with thickness of 0.3 mm. (**c**) Schwarz P with thickness of 0.1 mm. (**d**) Schwarz P with thickness of 0.3 mm.

Figure 3 shows some of the pictures taken by the optical microscope for the gyroid- and Schwarz P-type scaffolds. It is possible to see the different pore sizes and wall/strut thicknesses that characterize the geometry of scaffolds.

**Figure 4:**
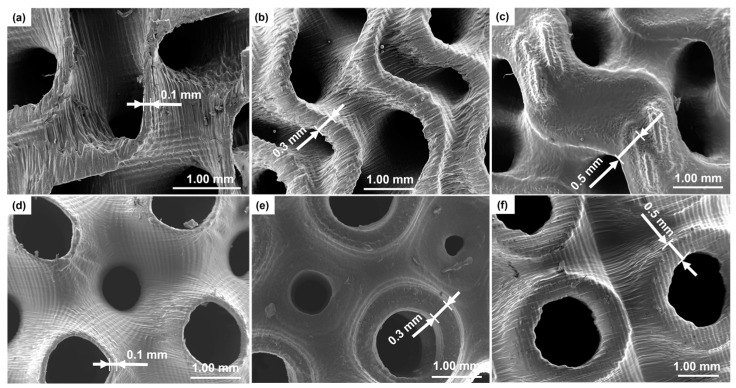
SEM images of TPMS scaffolds with a magnification of 22×. Gyroid type with a thickness of (**a**) 0.1 mm, (**b**) 0.3 mm, and (**c**) 0.5 mm; Schwarz P type with a thickness of (**d**) 0.1 mm, (**e**) 0.3 mm, and (**f**) 0.5 mm.

More microstructural details of the scaffolds were obtained by SEM; the results are displayed in Figure 4.

SEM analysis allows investigation of the sample morphology with better resolution than the digital optical microscope. The pore diameter and thickness of each scaffold were calculated from SEM images and compared with those obtained by the optical microscope measurements. Both techniques (SEM and digital optical microscopy) yielded comparable results in terms of wall thickness of gyroid- and Schwarz P-type scaffolds.

Quantification of printing fidelity was assessed by comparing the theoretical values set in Python and the actual values measured on 3D-printed samples. In both types of scaffolds with a thickness of 0.4 mm and 0.5 mm, the error on the programmed thickness was less than 10%. As regards the thinner scaffolds, the measurements were not so accurate because the edges were not clearly distinguishable: thus, the measurements had an error of 10–30%.

Pore sizes obtained for the Schwarz P structures were in a range of 600–1200 μm, which is similar to that observed in spongy bone [51]. As regards gyroid structures, the pore sizes measured by SEM were in a range of 500–600 μm for thickness of 0.1–0.2 mm and around 1100 μm for samples with a thickness of 0.3–0.5 mm.

**Figure 5:**
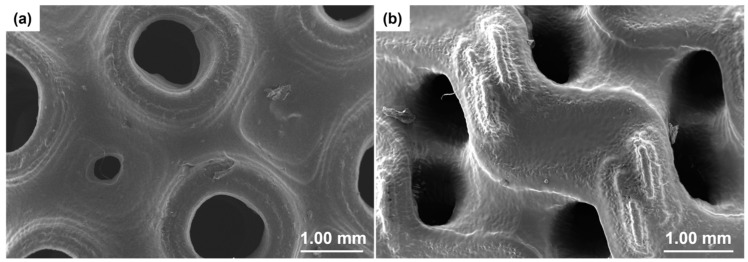
SEM images of TPMS structures with a thickness of 0.5 mm: (**a**) Schwarz P type and (**b**) gyroid type.

Comparing the images obtained from scaffolds with different thicknesses, structural variations can be observed in the morphology. Scaffolds with a thickness of 0.5 mm exhibited a smooth, compact surface compared to scaffolds with lower thickness. At the same time, as it is possible to note in Figure 5, the samples with 0.5 mm thickness exhibited some pore occlusions of the structure. This may be due to the fact that the wall thickness was too high compared to the unit cell size and this did not allow the creation of all pores set at the design stage.

Experimentally assessed values of density, relative density and porosity for TPMS scaffolds are collected in Table 2 and Table 3.

A certain trend could be noted for the various types of elementary cell length. For example, as the thickness of the lattice increased, the density of the lattice increased too, because there was more material composing it in spite of porosity.

The TPMS structures developed showed a porosity between 87 and 40 vol.%; this range of porosity is within the typical one that characterizes human spongy bone (50–90 vol.%) [52]. In particular, scaffolds with a unit cell length of 4 mm showed a porosity about 10% higher than the scaffold with a 3 mm unit cell length, despite having similar gross dimensions.

### 3.2. Mechanical Results

**Figure 6:**
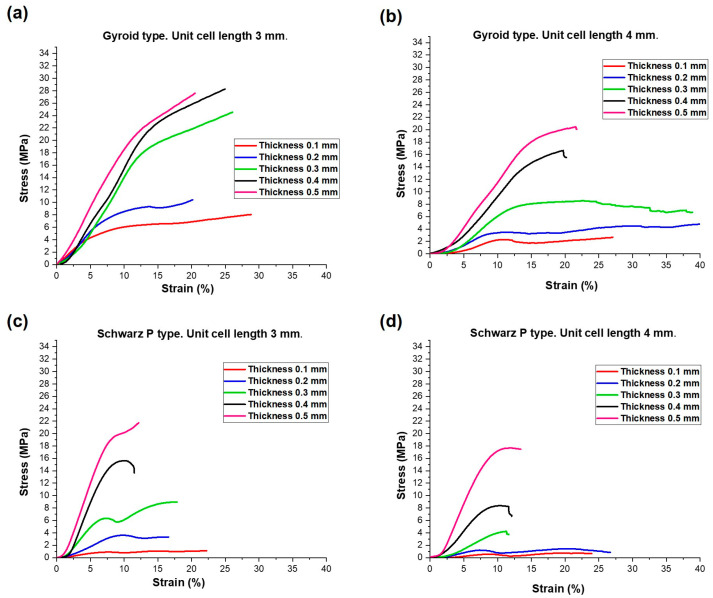
Compressive stress–strain behavior obtained for (**a**,**b**) gyroid-type and (**c**,**d**) Schwarz P-type scaffolds.

Some typical stress–strain curves obtained from the compressive tests for the gyroid- and Schwarz P-type scaffolds are reported in Figure 6. Five samples from every different class of scaffold thickness were tested. In the graphs, only one representative curve was plotted for each thickness type, and a comparison with scaffolds of the same type but having other thicknesses is provided.

**Figure 7:**
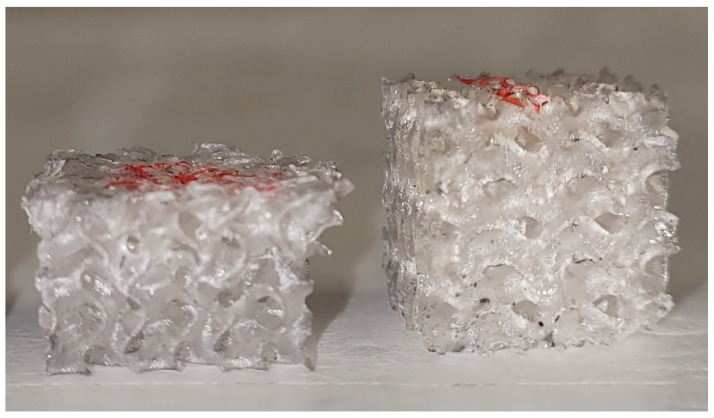
Gyroid-type scaffold before (**right**) and after the compression test (**left**).

There is a repeatable trend that characterizes stress–strain curves of scaffolds when they were subjected to compression tests. As displayed in the graphs, both scaffold types subjected to compression exhibited a mechanical behavior that was initially characterized by linear elastic deformation, followed by non-linear elastic deformation until the point of yield stress is achieved. From this point onwards, plastic deformation took place along with the rupture of internal parts of the scaffold. No specimens were completely broken during the compressive tests; after the elastic deformation, micro-breaking of the inner part of the lattice occurred immediately. An example of scaffold before and after the compression test (plastic collapse of polymeric walls) is shown in Figure 7.

The mechanical properties extracted from the stress–strain curves are reported in Table 4 and Table 5.

The values of Young’s modulus were found to decrease as the scaffold porosity increased, in perfect agreement with theoretical expectations. Furthermore, given that finding a relationship between scaffold geometry and mechanical properties is a goal, it can be observed that Young’s modulus has an increasing trend with increasing thickness. Overall, Young’s modulus values obtained in the compressive tests (10–320 MPa) are in the range of the elastic modulus of human spongy bone (10–500 MPa) [51].

**Figure 8:**
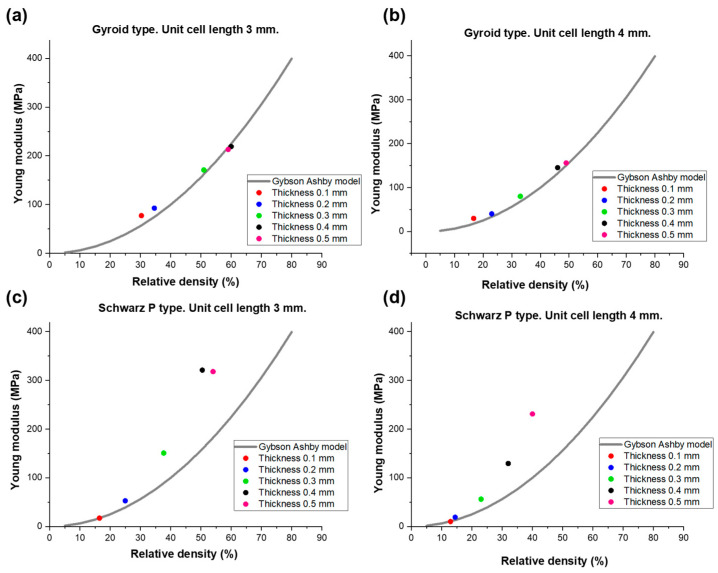
Relationship between Young’s modulus and relative density in (**a**,**b**) gyroid-type and (**c**,**d**) Schwartz P-type scaffolds. Experimental data (values of Young’s modulus and relative density) for each thickness and typology (circle) were interpolated by using the classical Gibson–Ashby power law reported in Equation (5) (continuous line).

The elastic modulus and compressive strength of scaffolds are expected to depend on both the constitutive properties of the solid material (i.e., non-porous “skeleton”) and the relative density of the porous samples. Under an early approximation, the TPMS scaffolds analyzed in the present work can be described as open-cell foams. In these cases, the relationship between mechanical properties and relative density is well fitted by power-law models. Specifically, the classical power law developed by Gibson and Ashby was used to describe the relationship between the elastic modulus and relative density of porous scaffolds [53]:

E = E_0_φ^2^ (5)

where E and φ (=ρ_s_/ρ_0_) are Young’s modulus and relative density, respectively, calculated for each scaffold, and E_0_ is the value of Young’s modulus of the solid part of the scaffolds (i.e., non-porous polymeric resin). In this regard, the value of E_0_ = 623.9 MPa was estimated by means of compressive tests on cuboids of resin with the same settings of the compressive tests of the porous scaffolds. The result of the data fitting is illustrated in Figure 8.

The results of the fitting using the Gibson–Ashby power-law model seem very suitable to describe the relationship between Young’s modulus and relative density for the gyroid-type scaffolds, obtaining a coefficient of determination of R^2^ = 0.97 for unit cell length equal to 3 mm and R^2^ = 0.96 for unit cell length equal to 4 mm. On the contrary, in the case of Schwarz P-type scaffolds, R^2^ = 0.41 and R^2^ = 0.35 were obtained for unit cell lengths of 3 and 4 mm, respectively. Figure 7 shows that Schwarz P-type scaffolds exhibit significantly higher values of Young’s modulus than those expected from the Gibson–Ashby model; this might be related to the shape of the pores (cells) being well aligned with the stress, which limits the shear stress within the construct. This suggests that other models could be more suitable for describing the relationship between elastic modulus and relative density for these structures; in this regard, the models developed by Pabst and Gregorova [54], Warren and Kraynik [55], and Zhu et al. [56] were also applied to interpolate these data, but the results of data fitting were found to be unsatisfactory in all cases (R^2^ = 0).

Gibson and Ashby also proposed a more general form of the power-law model, reported in Equation (5), to describe the relationship between elastic modulus and relative density, suggesting that for relative densities larger than 20% [57], it can be rewritten as:

(6) $$\frac{E}{E_{0}}=C\varphi^{n}$$

where C is an interpolating constant and *n* ≠ 2.

Values of *n* in the range of 1.3 to 3 were found for various porous microstructures [58], indicating a more complex dependence than typically obtained for periodic cell theories and pure bending of solid struts (in this case, *n* = 2 as in Equation (5)).

**Figure 9:**
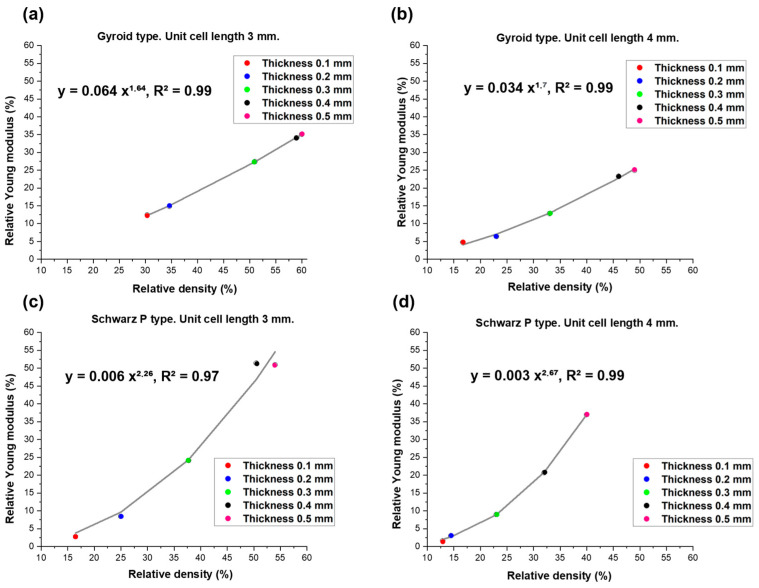
Relationship between relative Young’s modulus and relative density in (**a**,**b**) gyroid-type and (**c**,**d**) Schwartz P-type scaffolds. Experimental data (values of relative Young’s modulus and relative density) for each thickness and typology (circle) were interpolated by using the model described in Equation (6) (continuous line).

The results of data fitting are reported in Figure 9. Equation (6) is definitely more suitable than Equation (5) for the fitting of the Schwartz P-type scaffold data (R^2^ > 0.97), and yields even better predictions in the case of gyroid-type scaffolds. This second observation is consistent with previous findings reported by Maconachie et al. [59], who successfully applied such an equation type to acrylonitrile butadiene styrene (ABS) gyroid lattice structures manufactured by fused deposition modeling.

## 4. Conclusions

In this study, polymeric scaffolds with TMPS surfaces (Schwarz P and gyroid) were fabricated by using stereolithography in an attempt to reproduce the mechanical and structural characteristics of cancellous bone. The first phase of the study was dedicated to the creation of the scaffold structure through 3D modeling, and the measurements taken on the 3D-printed samples by SEM confirmed the reliability and repeatability of the fabrication method. In fact, the calculation of the elementary cell length did not differ more than 1.1% from those programmed in Python. This error corresponds to a length deviation of 20–30 μm, which may be due to measurement errors in the human–machine interface. The stereolithographic technique was useful in producing high-precision scaffolds with small, complex internal features and a relatively smooth surface finish. According to the dimensional analysis of the pores, the average pore size was even greater than those required for bone application, especially for the gyroid structure. However, the structures developed in this work showed a degree of porosity of 40–85 vol.%, which is comparable to the typical range of spongy bone.

Results from uniaxial compressive tests revealed that Young’s modulus and compressive strength are suitable for bone-contact applications. In general, gyroid scaffolds are less porous than Schwarz P ones, with the same value of wall thickness, and consistently are mechanically stronger. Data fitting also showed that the relationship between Young’s modulus and relative density φ (or alternatively, total porosity 1 − φ) for gyroid scaffolds could be well described by a second-order power law (classical Gibson–Ashby model for foam-like materials), while a more general power-law function can approximate the behavior of both scaffold types.

Further analyses of the biological behavior of the scaffolds deserve to be performed in the future in order to assess the interaction between porous biomaterials and cells, as well as how/to what extent the pore size and structural characteristics are able to favor cell attachment and promote cell proliferation. Future studies on other types of TPMS structures to extend the current results also deserve to be carried out.

## Figures and Tables

**Table 1:** Scaffold parameters set in Python.

Unit Cell Length (mm)	Constant Thickness (mm)	Theoretical Volume (mm^3^)
3	0.1	12 × 12 × 12
3	0.2	12 × 12 × 12
3	0.3	12 × 12 × 12
3	0.4	12 × 12 × 12
3	0.5	12 × 12 × 12
4	0.1	12 × 12 × 12
4	0.2	12 × 12 × 12
4	0.3	12 × 12 × 12
4	0.4	12 × 12 × 12
4	0.5	12 × 12 × 12

**Table 2:** Density and porosity values of scaffolds with a unit cell length of 4 mm.

	Thickness (mm)	Density (kg/m^3^)	Relative Density (%)	Porosity (vol.%)
Gyroid	0.1	195.1	16.7	83.3
	0.2	268.2	22.9	77.0
	0.3	379.1	32.4	67.6
	0.4	539.9	46.2	53.8
	0.5	574.5	49.2	50.9
Schwarz P	0.1	150.9	12.9	87.0
	0.2	168.9	14.5	85.6
	0.3	271.9	23.3	76.7
	0.4	369.8	31.6	68.4
	0.5	476.3	40.7	59.3

**Table 3:** Density and porosity values of scaffolds with a unit cell length of 3 mm.

	Thickness (mm)	Density (kg/m^3^)	Relative Density (%)	Porosity (vol.%)
Gyroid	0.1	354.5	30.3	69.7
	0.2	404.3	34.6	65.4
	0.3	605.4	51.8	48.2
	0.4	706.4	60.4	39.6
	0.5	690.6	59.1	40.9
Schwarz P	0.1	192.4	16.5	83.5
	0.2	291.9	24.9	75.0
	0.3	441.5	37.8	62.2
	0.4	589.6	50.4	49.6
	0.5	631.7	54.1	45.9

**Table 4:** Mechanical properties of porous scaffold with a unit cell length of 4 mm.

	Thickness (mm)	Young’s Modulus (MPa)	Compressive Strength (MPa)
Gyroid	0.1	29.7 ± 7.8	1.7 ± 0.6
	0.2	39.9 ± 9.0	3.1 ± 1.1
	0.3	80.4 ± 11.4	5.6 ± 1.5
	0.4	145.3 ± 25.0	13.8 ± 2.5
	0.5	156 ± 10.3	17.7 ± 1.8
Schwarz P	0.1	10.1 ± 1.5	0.5 ± 0.1
	0.2	19.1 ± 9.2	1.1 ± 0.3
	0.3	56.3 ± 11.9	3.6 ± 0.1
	0.4	129.3 ± 33.5	7.9 ± 1.0
	0.5	231 ± 41.9	11.9 ± 1.5

**Table 5:** Mechanical properties of porous scaffold with a unit cell length of 3 mm.

	Thickness (mm)	Young’s Modulus (MPa)	Compressive Strength (MPa)
Gyroid	0.1	77.5 ± 25.7	3.3 ± 0.3
	0.2	92.7 ± 27.5	5.3 ± 1.2
	0.3	171.0 ± 4.1	14.9 ± 0.1
	0.4	219.2 ± 32.1	22.9 ± 4.3
	0.5	213.1 ± 21.1	20.1 ± 2.0
Schwarz P	0.1	17.2 ± 3.4	0.8 ± 0.1
	0.2	52.7 ± 23.3	2.9 ± 0.9
	0.3	150.7 ± 48.9	6.9 ± 2.0
	0.4	321.7 ± 25.0	15.5 ± 2.8
	0.5	317.9 ± 48.9	19.9 ± 1.9

## Data Availability

Data are included in the manuscript.
